# Cross-Situational Statistical Learning of New Words Despite Bilateral Hippocampal Damage and Severe Amnesia

**DOI:** 10.3389/fnhum.2019.00448

**Published:** 2020-01-14

**Authors:** David E. Warren, Tanja C. Roembke, Natalie V. Covington, Bob McMurray, Melissa C. Duff

**Affiliations:** ^1^Department of Neurological Sciences, University of Nebraska Medical Center, Omaha, NE, United States; ^2^Institute of Psychology, RWTH Aachen University, Aachen, Germany; ^3^Department of Hearing and Speech Sciences, Vanderbilt University, Nashville, TN, United States; ^4^Psychological and Brain Sciences, University of Iowa, Iowa, IA, United States

**Keywords:** word learning, amnesia, hippocampus, cross-situational statistical learning, statistical learning, declarative memory, relational memory

## Abstract

Word learning requires learners to bind together arbitrarily-related phonological, visual, and conceptual information. Prior work suggests that this binding can be robustly achieved *via* incidental cross-situational statistical exposure to words and referents. When cross-situational statistical learning (CSSL) is tested in the laboratory, there is no information on any given trial to identify the referent of a novel word. However, by tracking which objects co-occur with each word across trials, learners may acquire mappings through statistical association. While CSSL behavior is well-characterized, its brain correlates are not. The arbitrary nature of CSSL mappings suggests hippocampal involvement, but the incremental, statistical nature of the learning raises the possibility of neocortical or procedural learning systems. Prior studies have shown that neurological patients with hippocampal pathology have word-learning impairments, but this has not been tested in a statistical learning paradigm. Here, we used a neuropsychological approach to test whether patients with bilateral hippocampal pathology (*N* = 3) could learn new words in a CSSL paradigm. In the task, patients and healthy comparison participants completed a CSSL word-learning task in which they acquired eight word/object mappings. During each trial of the CSSL task, participants saw two objects on a computer display, heard one novel word, and selected the most likely referent. Across trials, words were 100% likely to co-occur with their referent, but only 14.3% likely with non-referents. Two of three amnesic patients learned the associations between objects and word forms, although performance was impaired relative to healthy comparison participants. Our findings show that the hippocampus is not strictly necessary for CSSL for words, although it may facilitate such learning. This is consistent with a hybrid account of CSSL supported by implicit and explicit memory systems, and may have translational applications for remediation of (word-) learning deficits in neurological populations with hippocampal pathology.

## Introduction

Statistical learning is the ability to learn from repeated (often incidental) exposure to probabilistic associations among elements of the input (Frost et al., 2019). This form of learning has been long-studied in the language literature, and it is posited to be particularly important for very early cognitive development of language (Saffran et al., 1996; Smith and Yu, 2008) as well as other domains. In language development, substantial learning occurs in complex environments that require segmentation of continuous input based on repeated exposure and probabilistic associations (Saffran et al., 1996; Karuza et al., 2013). Studies of infants, children, and adults suggest that statistical learning can occur at multiple developmental stages and can support learning at multiple levels of language (speech perception, word recognition, syntax, etc.; Saffran et al., 1996; Conway and Christiansen, 2005; Yu and Smith, 2007; Baldwin et al., 2008; Schapiro et al., 2016).

Recently, statistical learning has received attention in the memory literature (Schapiro et al., 2014; Covington et al., 2018). This attention has prompted new descriptions of the empirical phenomenon of statistical learning in terminology of *multiple memory systems*. The multiple memory systems perspective suggests that several unique brain systems support different types and rates of learning (Cohen and Squire, 1980; McClelland et al., 1995; Eichenbaum and Cohen, 2001; Norman and O’Reilly, 2003; Ranganath, 2010). Which of these systems support statistical learning? Novel findings from neuropsychological investigations indicate that certain domain-specific forms of statistical learning may (or may not) rely on memory processes associated with the medial temporal lobe and hippocampus (Schapiro et al., 2014; Covington et al., 2018). However, prior neuropsychological investigations have not tested statistical learning of multimodal associations. This is important because learning multimodal associations such as the mappings between new words and their referents (i.e., word learning) may span multiple learning systems. Further, the necessity of specific memory systems (and associated brain regions) for statistical learning of linguistic information such as words has not been evaluated.

### Statistical Learning and Multiple Memory Systems

Until very recently, statistical learning has been primarily an empirical phenomenon with an ambiguous relationship to theories of memory systems. Learning in a statistical context requires learners to extract consistent regularities (statistical associations) from repeated exposure to complex input which contains more than one element. Statistical learning has import for memory theory because the learned representations cannot be trivially categorized into a single type of memory representation described by theories of multiple memory systems.

Theories positing multiple memory systems were developed in part to address findings from neuropsychological studies of amnesic patients with damage to the medial temporal lobe (Scoville and Milner, 1957; Cohen and Squire, 1980). These theories suggest that (at least) two types of memory representations are supported by unique brain correlates. Under this framework, *procedural* (or *non-relational*) memory stores information about individual elements of prior experience incrementally and in a manner that supports future expression under primarily *implicit* conditions (e.g., faster response times, increased sensitivity, or experience-dependent response bias). *Declarative* (or *relational-declarative*) memory stores information about relations between elements of prior experience rapidly and in a manner that supports future expression primarily under *explicit* conditions (e.g., free recall, old/new recognition, or multiple-choice recognition). Critically, neuropsychological studies indicate that the medial temporal lobes—including the hippocampus—are necessary for normal declarative-relational memory but not procedural memory (Scoville and Milner, 1957; Cohen and Squire, 1980; McClelland et al., 1995; Poldrack et al., 2001).

We note that the term “relational” has been used in psychology and neuroscience to describe various forms of representation (Eichenbaum and Cohen, 2001; Hummel and Holyoak, 2003; Cleland et al., 2007). Here, we use “relational” as discussed by Eichenbaum et al. (1992) who observed that the “… the critical property of declarative [relational] memory … is the encoding of memories in terms of the relations among multiple items …” (p. 3). In describing laboratory tests of relational memory, those authors noted that “[i]n some formal tests of memory, such as paired associate learning, demands for relational representation and/or representational flexibility—and hence declarative [relational] memory—are immediately evident” (p. 7; emphasis added).

In statistical learning, the incremental and incidental (i.e., implicit) acquisition of statistical associations between items strongly resembles the pace and function of procedural learning and representations. At the same time, statistical learning has also recently been studied in the context of learning mappings between words and objects (Yu and Smith, 2007; Smith and Yu, 2008; Roembke and McMurray, 2016; Roembke et al., 2018). This type of mapping requires that participants learn and express arbitrary relations (e.g., between faces and scenes, among sets of novel objects, or associations between words and referents), and relational representation is thought to rely on hippocampal-dependent relational-declarative representations (Eichenbaum et al., 1994; Eichenbaum and Cohen, 2001; Davachi and Dobbins, 2008; Ranganath, 2010). Because statistical word learning involves the incremental acquisition of arbitrary relations, describing the phenomenon using the terminology of multiple memory systems is challenging. This suggests that statistical learning paradigms requiring acquisition of arbitrary relations—such as word-referent learning—may provide novel opportunities to test and extend theories of multiple memory systems.

### Cross-Situational Statistical Learning and Multiple Memory Systems

Evidence for statistical forms of word learning comes from the cross-situational statistical learning (CSSL) paradigm. In this paradigm, participants see an array of unfamiliar objects while hearing one or more novel word forms. Initially, the word-referent mapping appears completely random—there is no information to lead the learner to the correct referent. However, across trials, a given word is more likely to be heard with its referent than other objects. Hence, by tracking the co-occurrence between word forms and referents (objects), learners can acquire the mappings. This simple manipulation of statistical co-occurrence is sufficient to drive robust memory for word-referent pairings. Laboratory studies using this paradigm suggest that infants and adults can learn word-referent pairings from their environment through purely implicit statistical exposure (Saffran et al., 1996; Yu and Smith, 2007; Smith and Yu, 2008; Roembke and McMurray, 2016; Roembke et al., 2018).

CSSL of word-referent mappings has been hypothesized to be supported by various cognitive mechanisms. One hypothetical mechanism is gradual and associative: learners track associations between each word and multiple referents, and these associations reflect the relative evidence for a given mapping (Roembke and McMurray, 2016). An alternative instead relies on “single informative exposures”; here, learners form only a single hypothesis for a word’s referent, and update or reject this hypothesis during subsequent trials using inferential processes (Trueswell et al., 2013). Importantly, these hypothesized mechanisms need not be mutually exclusive and could function in parallel (Yurovsky and Frank, 2015). These cognitive mechanisms could be roughly mapped to components of the multiple memory systems framework. That is, the more gradual associative form of learning could be primarily mediated by non-hippocampal/non-relational systems, whereas the more inferential hypothesis-testing scenario could be supported by the hippocampal-relational system.

Note that in the CSSL paradigm, the association between the sound of a word and its referent is overwhelmingly an arbitrary relation. Yet, thousands of these arbitrary mappings are mastered by children during healthy language development (Bloom, 1973). According to one multiple memory systems perspective, learning about arbitrary associations between items is exclusively the domain of hippocampal-dependent relational-declarative memory (Eichenbaum and Cohen, 2001; Davachi and Dobbins, 2008; Ranganath, 2010). Under this theory—which challenges both the gradual associative account of learning and the hybrid account—a reasonable hypothesis would be that CSSL of new word-referent associations requires the hippocampus and will, therefore, be impaired in patients with hippocampal pathology. However, another possibility is that word-referent associations can be learned at least in part *via* statistical mechanisms with non-hippocampal brain correlates, and this would yield spared learning in patients with hippocampal pathology. Thus, the arbitrary nature of the mapping problem makes cross-situational statistical word learning a unique paradigm in which the contributions of multiple memory systems to statistical learning can be evaluated.

### Brain Correlates of Statistical Learning

Evidence from neuroimaging and neuropsychology is mixed regarding potential contributions of medial temporal lobe regions to any form of statistical learning. Functional neuroimaging with fMRI has shown hippocampal activation during statistical learning of sequential dependencies in healthy young adults (Turk-Browne et al., 2009; Schapiro et al., 2016). Consistent with this, Schapiro et al. (2014) used a neuropsychological approach to study statistical learning of sequential dependencies in a patient with extensive medial temporal lobe damage (including the hippocampus). The patient performed at chance, and her performance was impaired relative to healthy comparison participants, suggesting that the medial temporal lobe may be necessary for statistical learning. Interpretation of these findings must be tempered, however, by results from a larger group of amnesic patients including some with focal hippocampal pathology (Covington et al., 2018). In that study, healthy comparison participants showed greater statistical learning than patients with focal hippocampal pathology. However, the patients still showed evidence of statistical learning that was above chance and often within the lower extent of the healthy range. Taken together, findings from previous studies suggest that the hippocampus may contribute to—but not be necessary for—statistical learning.

Previous neuropsychological studies of statistical learning have principally focused on sequential temporal dependencies among unimodal elements (syllables, tones, symbols, etc.). CSSL for words has not been examined in patients with hippocampal pathology. Critically, this form of feedback-free learning is arbitrary, temporally spaced, and multimodal—properties that may be consistent with hippocampus-dependent relational representations.

The current study is the first to explicitly test whether the hippocampus is necessary for CSSL. A role for the hippocampus in CSSL may have special relevance in early life (e.g., healthy development of language and vocabulary) and late-life (e.g., word-learning impairments in healthy and pathological aging). Relevant to this point, the hippocampus changes throughout life and both early development and late-life are periods when the hippocampus functions differently than in healthy maturity (i.e., young adulthood; Raz et al., 2004; Ghetti and Bunge, 2012; Ofen, 2012; Fjell et al., 2013). Prior work has established that the hippocampus is necessary for normal learning of word-referent mappings under certain explicit and implicit instructional regimes (Smith et al., 2014; Warren and Duff, 2014; but see Sharon et al., 2011). In contrast, Vargha-Khadem et al. (1997) reported results from children with perinatal or childhood hippocampal pathology who “… attained levels of speech and language competence, literacy, and factual knowledge … within the low average to average range.” This suggests that hippocampus is not strictly necessary for ecological word learning. A recent study by Berens et al. (2018) studied CSSL in neurotypical adults using functional MRI. They found evidence for a quick learning mechanism that is consistent with rapid pattern separation processes in the hippocampus. However, CSSL for words has not been tested neuropsychologically in adults with bilateral hippocampal pathology. This is essential for understanding the role of the hippocampus in different types of statistical learning (and for word learning more broadly).

Previous findings could support predictions for or against a hippocampal contribution to CSSL. Hippocampal amnesia has a profound negative impact on relational-declarative memory in general (Scoville and Milner, 1957; Cohen and Squire, 1980; Ryan et al., 2000; Hannula et al., 2006) and word learning specifically (Gabrieli et al., 1988; Postle and Corkin, 1998; Warren and Duff, 2014). Moreover, words exemplify the type of highly relational stimuli that require hippocampus (Warren and Duff, 2014). Prior studies of word learning by patients with hippocampal pathology have demonstrated that patients learn words more slowly and less successfully than healthy comparison participants under a variety of instructional conditions [e.g., explicit encoding (EE) and fast mapping; e.g., Warren and Duff, 2014]. These points suggest that hippocampus is necessary for normal CSSL.

However, CSSL paradigms are frequently implicit, and some studies have reported that the hippocampus is not necessary for word learning in implicit tasks (Sharon et al., 2011). Further, CSSL paradigms often employ a style of frequent repetition of stimuli that partly resembles procedural/non-declarative learning paradigms (e.g., errorless learning) in which patients with MTL or hippocampal pathology can learn as well as healthy comparisons participants (Scoville and Milner, 1957; Cohen and Squire, 1980; Glisky et al., 1986; Glisky, 1992).

These contrasting perspectives illustrate the ambiguous state of the current literature, and they motivate a targeted study of hippocampal necessity for word learning in a CSSL paradigm. Further, by studying a unique form of statistical learning, such findings might expand and inform debates over hippocampal necessity for statistical learning more generally.

### Current Study

Here, we used a neuropsychological approach to test the necessity of the hippocampus for CSSL in the domain of word learning. We adapted a CSSL task that has been previously reported (Roembke and McMurray, 2016) for our study. In this task, participants learn statistical associations between phonological and visual information across many presentations. On each trial, participants hear one novel phonological word form and view two novel objects. Each word form is consistently presented (across trials) with a specific object; the other object is a randomly-selected competitor (itself associated with a different word). Thus, the task requires learning a set of arbitrary relationships between phonological and visual stimuli in the presence of potentially interfering competitors. Damage to the hippocampus would be predicted to impair the relational memory abilities needed to learn such arbitrary relations. An EE task was also administered separately. The rationale for the EE task, which involved sequential exposure to word-referent associations without competitor items, was to measure simple (non-statistical) learning of arbitrary relations. Patient performance on each task was compared to healthy normal comparison participants.

Statistical learning of multi-modal associations is novel in this patient population. However, this study also expands on previous research studying other forms of statistical learning in patients with hippocampal pathology. In prior work (e.g., studying statistical learning of temporally adjacent dependencies), learning is assessed at a single time-point, in a two-alternative forced-choice (2AFC) post-test after the exposure phase (Schapiro et al., 2014; Covington et al., 2018). In contrast, we assess learning over time, and this will contribute to understanding the trajectory of statistical learning in the absence of hippocampal contributions.

## Materials and Methods

### Participants

Three groups of participants were recruited. First, we recruited a group of patients (*N* = 3) with hippocampal pathology (Table 1 and next paragraphs). All patients had participated in a prior study of hippocampal necessity for statistical learning (but not CSSL; Covington et al., 2018). Patients completed both a CSSL task and an EE task. Second, we recruited a group of healthy normal comparison participants (NC; *N* = 12) with no history of neurological or psychiatric disease. These were used as comparisons for the novel CSSL task. Each NC participant was matched to one of the patients for sex, handedness, age (±5 years), and education (±2 years); in total, four NC participants were matched to each patient. This matching strategy was selected to provide sufficient statistical power to detect deficits in performance in the patient group based on prior research in healthy adults (Roembke and McMurray, 2016). Finally, another smaller group of healthy normal comparison participants (*N* = 4) was recruited to complete the EE task (see below). As with the previous NC group, these participants were demographically matched to the patients (here, one-to-one). We had a strong *a priori* expectation that the massed practice of the EE condition would yield ceiling performance in NC participants (which was confirmed), so this second NC group was recruited principally for proof-of-method.

**Table 1:** Demographic and neuropsychological data characterizing participants with amnesia.

ID	Age	Sex	Edu.	Eti.	Chr.	Hand	FSIQ	VIQ	PIQ	DS	BNT	GMI	AVLT	CFT C/R	HcV
1846	52	F	14	An./SE	22	100	84	88	86	10	43	57	7/3	28/6	−4.23*
2363	59	M	18	An.	17	100	98	112	91	8	58	73	8/0	26/5	−2.64*
2563	61	M	16	An.	16	−80	94	91	98	14	52	63	10/4	36/7	NA

*Individual scores are presented for each patient with hippocampal pathology. The significant memory impairment of the amnesic group is evident in several neuropsychological measures. Abbreviations: Age, years; Edu., education, years; Chr., Chronicity, years since injury; Hand, handedness (+100 = fully right-handed, −100 = fully left-handed); Eti., Etiology; Anoxia/An., anoxic/ischemic episode, SE, status epilepticus; FSIQ, WAIS-III full-scale IQ (Weschler, 1997); VIQ, verbal IQ; PIQ, performance IQ; DS, WAIS 3/4 Digit Span; WMS-III GMI, general memory index (The Psychological Corporation, 1997); AVLT, Rey Auditory Verbal Learning Task, trial 5/30-min. delay; CFT, Complex Figure Task copy/recall (Rey, 1941; Osterrieth, 1944); BNT, Boston Naming Test (Goodglass et al., 1983); HcV, bilateral hippocampal volumes per Allen et al. (2006). Volumes are expressed in Studentized residuals relative to normative expectations: NA, volumetric measurements unavailable due to contraindication for MRI*.

Patients had severe, selective deficits in declarative memory according to neuropsychological assessments (Table 1). Impairment of declarative memory (including visual and verbal domains) was evident in patients’ profoundly impaired performance (≥2 SD below normal) on the WMS-III General Memory Index, Rey Auditory-Verbal Learning Task, and Rey-Osterrieth Complex Figure Task. Other cognitive abilities were generally preserved and in the normal range. Because naming abilities may be of special importance for word learning and CSSL, we also considered a neuropsychological measure of naming. Results of the Boston Naming Test indicated that naming performance was normal for patients 2363 and 2563 but impaired for patient 1846 (43/60, first percentile). However, 1846 performs normally when naming animals, fruits, and vegetables (Warren et al., 2012, p. 347). We interpreted 1846’s pattern of performance on naming tasks as evidence that her naming abilities were sufficiently well-preserved for her to participate in this study.

Patients had pathological bilateral atrophy of the hippocampus as confirmed by neuroimaging studies. Two patients (1846 and 2363) had substantial atrophy of the hippocampus confirmed with high-resolution T1-weighted MRI (Allen et al., 2006). In that report, the authors used previously established estimates of adult hippocampal volume (measured through manual tracing) from T1-weighted MRI data of healthy adults age 22–88 (Allen et al., 2005). Adjusted for age and sex based on a regression model fit to the normative data, the hippocampal volume of patient 1846 was 4.23 standard deviations below normal expectations (53% reduction); for patient 2363, hippocampal volume was 2.64 standard deviations below normal expectations (28% reduction). Patient 1846 was later studied with ultra-high-resolution T2-weighted MRI (Warren et al., 2012). Analysis of those data confirmed the earlier findings of hippocampal atrophy greatly exceeding expectations for age. The remaining patient (2563) wears a pacemaker and is contraindicated for MRI studies. His anatomy was instead visualized with computerized tomography and atrophy of the hippocampal region was reported (but not quantified) by an expert rater (Hannula et al., 2006).

Patients were recruited from the Iowa Registry of Neurological Patients. Comparison participants were recruited from Iowa City and surrounding communities. This research was approved by the University of Iowa Human Subjects Office and by the Biomedical Institutional Review Board, and the study was conducted according to the principles expressed in the Declaration of Helsinki. Informed consent was obtained from all participants prior to their first experimental session. Consent documents described the study’s purpose as follows: “… to investigate whether certain regions of the brain participate in the learning and expression of names.” All participants were remunerated at $15/h.

### Stimuli

**Figure 1:**
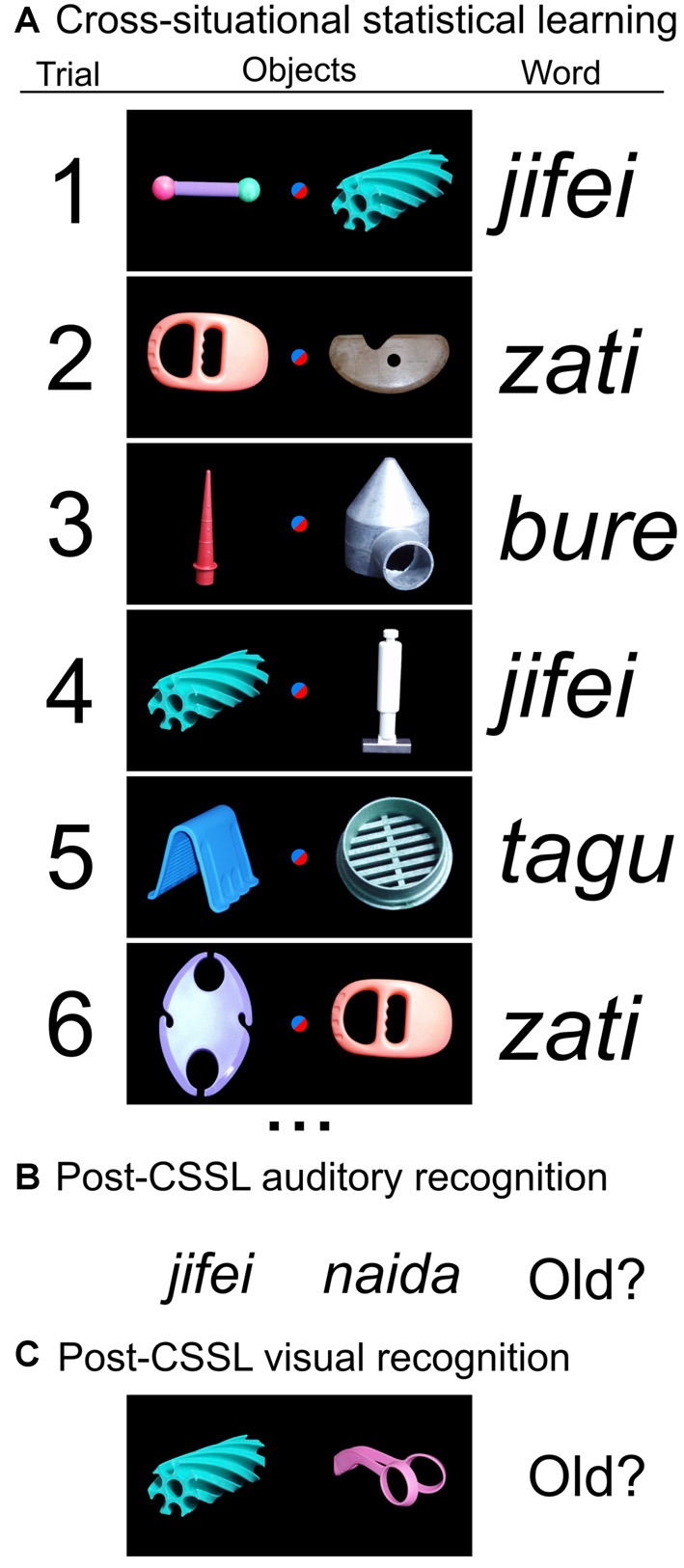
Procedure for cross-situational statistical learning (CSSL) and recognition testing. Our procedure adopted the approach of a previous study (Roembke and McMurray, 2016) to implement and test CSSL. **(A)** The procedure for cross-situational learning involved studying (auditory-visual) word-object pairs accompanied by a competitor object. The association of a specific word with a specific object (e.g., word *jifei* with the spiral blue object) was invariant across trials but not immediately obvious to participants because of the competitor object. Participants selected the object they believed was associated with the word to advance to the next trial. Eight word-object pairs were presented 14 times per block; three blocks were completed. **(B)** After the CSSL task, memory for the auditory word stimuli was tested using a two-alternative forced-choice (2AFC) recognition test. Two words (one studied, one novel) were presented auditorily in sequence, and the participant decided which had been studied. **(C)** Memory for the visual object stimuli was also tested using a 2AFC recognition test. Two objects (one studied, one novel) were presented on the display, and the participant decided which had been studied.

Materials were auditory and visual stimuli that have been previously described (Roembke and McMurray, 2016). Visual stimuli were novel visual objects superimposed on a black background (Figure 1). Auditory stimuli were two-syllable, consonant-vowel-consonant-vowel (CVCV) pseudowords which were phonologically legal in English. There was no phonological overlap among any words at the onset. Words were recorded by a native speaker of English, and five tokens of each word were used to include natural variability in the phonological representation of the word. All materials were pre-experimentally unfamiliar to participants.

### Equipment

Visual stimuli were presented on a 21-in LCD monitor (Multi-Sync 2190UXi, NEC Corporation of America, Irving, TX, USA) at a distance of 550 mm. Behavioral responses were made with a computer mouse. During the tasks, subjects placed their head in a padded chinrest/headrest apparatus, and eye movements were monitored at a sampling rate of 1,000 Hz using an EyeLink 1000 remote infrared camera system (SR Research Limited, Kanata, ON, Canada). Calibration procedures were conducted every 30 trials and ensured that gaze position was accurate to within 1° of visual angle.

### Procedure

#### Cross-Situational Statistical Learning

Participants completed a set of tasks designed to test CSSL of words (Figure 1). Our procedure was similar to that of Roembke and McMurray (2016). There were three phases. First, participants completed a learning phase in which visual and auditory stimuli were presented; each learning trial required a response for learning assessment. Second, memory for the auditory word forms was tested using a 2AFC format. Third, memory for the visual stimuli was tested using a 2AFC format. Visual and auditory stimuli were unfamiliar to the participants prior to the experiment.

During the CSSL phase, the participant was told that their task was to learn which visual stimulus (“object”) was paired with which auditory stimulus (“word”). During each trial, two objects were presented along with one word (Figure 1A); the participant was instructed to select the object associated with the word. Participants were told that initially their selections would be guesses, but they should learn the associations over time. The experimenter ensured that all participants understood the instructions before testing began.

During each trial, two objects were presented on the left and right sides of the display with a blue dot in the center. Participants were required to fixate the dot to continue. After 1,050 ms, the blue dot turned red signaling the participant to click the dot. Clicking the red dot then triggered the presentation of the word. After hearing the word, the participant clicked on one of the objects to advance to the next trial. No feedback was provided following the response. The referent associated with the word was presented equally often in the left and right positions across trials. For the patients, the experimenter checked between blocks and as needed to ensure that patients’ understanding of the instruction set was maintained throughout testing.

Within the CSSL phase, eight word-image pairs were presented 14 times each per block; three blocks were administered. Word-image pairs were unique for each patient (and their matched NC participants). By design, the word presented during each trial was uniquely associated with one of the objects, and the association was deterministic (i.e., a given word was exclusively presented in the presence of its paired object). The second, competitor object was selected at random from the non-paired objects. The random selection was made without replacement to avoid unintentional statistical association with a word; thus, the co-occurrence of each word with each non-paired object was one in seven (14.3%) vs. 100% for the paired object.

After the learning phase, two recognition tests were administered. In the auditory recognition test, the participant was asked to identify which of two words had been presented during the learning phase. The target was a word from the learning phase; the competitor item was intraexperimentally novel but otherwise had similar stimulus properties to words from the CSSL phase. The two words were presented sequentially separated by a short, silent pause. Simultaneously with each word, a colored square (orange and blue for the two words, respectively) appeared. The participant was instructed to tell the experimenter which item was studied, and the experimenter recorded the response. The interactive display allowed the participant to replay either word *ad-lib* (by clicking the orange or blue square) until a decision had been made. Once the participant’s response was recorded, the trial was advanced to another auditory recognition trial until all words from the learning phase had been tested. The target word was presented equally often in the first and second (and thus, left and right) positions.

The visual recognition phase followed a similar logic. The participant was asked to identify which one of two objects had been presented during the learning phase. The target was an object from the learning phase; the competitor item was intraexperimentally novel but had similar stimulus properties. The two objects were presented simultaneously on the display at the left and right sides of the display. The participant observed the test display, then responded by clicking on one object (the studied object) with the mouse, thus advancing the trial. All objects from the learning phase were tested using this approach. The target item was presented equally often in the left and right positions.

All patients with hippocampal pathology completed the CSSL task along with 12 NC participants (matched 4:1 as described above).

#### Explicit Encoding

To contrast with the CSSL task, we also administered an EE task. In the EE task, each trial presented a single unfamiliar visual stimulus (object) along with a single unfamiliar auditory stimulus (word). To advance to the next trial, participants clicked the mouse on the (single) object. As in the CSSL condition, no feedback was provided during learning exposures. After 28 exposures to each of eight word-object pairs (14 presentations/block × 2 blocks), participants completed a 2AFC recognition test which matched the format of CSSL learning blocks (8 word-object pairs × 7 presentations = 56 trials). No feedback was provided during the 2AFC recognition test. All patients with hippocampal pathology completed the EE task along with four task-naïve NC participants (matched 1:1 as described above). Importantly, stimuli presented during the EE and CSSL tasks were unique and not overlapping (thus, word-referent learning during the CSSL task did not influence EE performance). For patients, the EE task was always administered after the CSSL task and in a separate test session.

### Analysis

Data were aggregated using Python 3.6 and Python’s panda’s module. Data were analyzed and visualized using R 3.5.1 and the lme4, afex, psycho, and ggplot2 libraries. All statistical tests used *α* = 0.05 to determine statistical significance. This value was corrected for multiple comparisons in cases described below, and this correction is indicated with *p*_c_ in the Results.

Data from the CSSL learning phase were analyzed to assess group and individual trends in accuracy across learning exposures. Specifically, the 336 total trials (8 word-object pairs × 14 presentations/block × 3 blocks) for each participant were divided into six sequential epochs of 56 trials each. Performance during each epoch was operationalized as the proportion of trials in which the participant selected the object paired with the word in our experimental design. This proportion correct (prop. correct) measure for each participant in each epoch is plotted to illustrate performance by block.

First, we tested whether the NC group showed a learning trend across time and whether NC participants matched to different patients performed differently. This was analyzed using a generalized linear mixed-effects model with a binomial link function as implemented in the R package/function afex::mixed. The model had fixed effects for learning epoch (factor, levels: 1–6) and matched patient (factor, levels: 1846, 2363, and 2563); the participant was a random effect (intercept). β weights for factor levels were tested for statistical significance with the likelihood ratio method; statistically significant differences among factor levels were tested with a chi-squared (*χ*^2^) statistic.

Second, we tested whether patient performance differed from NC performance using a Bayesian implementation of Crawford’s modified *T*-test (Crawford and Garthwaite, 2007). This was applied at each epoch; we corrected *α* for this test using Bonferroni’s method with a correction factor of six (i.e., *α* = 0.05/6 = 0.0083); Bonferroni-corrected tests are indicated with *p*_c_.

Third, we tested whether the patient performance was greater than chance (prop. correct = 0.5) using a one-sided binomial test; this test was corrected for multiple comparisons as before.

Data from the auditory and visual recognition tests were analyzed to test group differences in recognition performance after CSSL exposure. When sufficient variance was available in the NC group, we used Crawford’s modified *T*-test to assess whether patients performed in the NC range. Also, we tested whether the patient performance was greater than chance (prop. correct = 0.5) using a one-sided binomial test.

Data from the EE task were analyzed to test group differences in learning after EE exposure. When sufficient variance was available in the NC group, we used Crawford’s modified *T*-test to assess whether patients performed in the NC range. Also, we tested whether the patient performance was greater than from chance (prop. correct = 0.5) using a one-sided binomial test.

## Results

**Figure 2:**
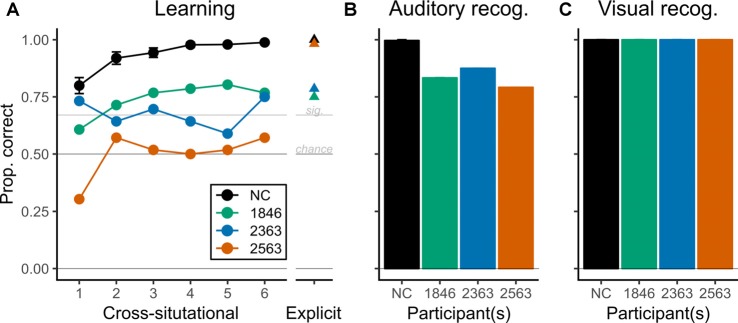
Performance during CSSL and recognition. Patients with hippocampal pathology showed evidence of CSSL for words that was above chance but reduced relative to comparison participants. Note that the ordinate (Proportion correct) is common to all panels. **(A)** The healthy normal comparison group (NC) showed improvements in proportion correct across CSSL epochs as expected based on prior work (Roembke and McMurray, 2016). Two patients (1846, green, and 2363, blue) also showed significant, above-chance performance during the CSSL task (thresholds for chance and statistical significance are represented with horizontal lines). However, their performance was less than the NC group, especially in later epochs. Patient 2563 performed at chance throughout. Whiskers represent SEM for the NC group. **(B)** Recognition for words (auditory) was above chance for all participants, but the patients recognized fewer words than the NC group. **(C)** Recognition for objects (visual) was perfect for all participants.

In the CSSL task, two of three patients with hippocampal pathology showed above-chance performance for word-object associations by the final epoch, but performed less well than the NC group (Figure 2A); the third patient did not show evidence of learning.

Specifically, the NC group showed no differences by matched patient, $\chi_{\left(2\right)}^{2}$ = 3.889, *p* = 0.143, but performed above chance in each epoch (each *T*_(11)_ > 8.5, each *p*_c_ < 0.001) and showed differences in accuracy across epochs, $\chi_{\left(5\right)}^{2}$ = 253, *p* < 0.001, such that performance increased monotonically (Figure 2A, black line). With no evident differences by matched patient, the NC group was combined for all subsequent analyses.

Similar to the learning trend observed in the NC group, patient 1846 showed monotonically increasing performance across the first five epochs and performed statistically better than a chance for epochs 2–6 (each p_c_ < 0.001). Although her performance was not statistically different from the NC group in epochs 1–3 (each *p*_c_ > 0.01), her performance was significantly less than the NC group in epochs 4–6 (each *p*_c_ < 0.001). Patient 2363 also showed learning but presented a less consistent pattern of performance. He performed statistically above chance in epochs 1, 3, and 6 (each *p*_c_ < 0.0025) and had performance statistically less than the NC group in all but the first epoch (each *p*_c_ < 0.01 for epochs 2–6). Finally, patient 2563 showed no significant evidence of any learning during the CSSL task: he never performed above chance (each *p*_c_ > 0.175), and his performance was always less than the NC group (each *p*_c_ < 0.0025). To reiterate, two of three patients showed evidence of learning word-object associations during the CSSL task while the third did not.

Auditory and visual recognition performance after CSSL exposure suggested that patients retained the knowledge of the individual studied stimuli, although recognition performance relative to the NC group diverged by modality. In the auditory recognition task, the NC group was effectively at the ceiling—11 of 12 NC participants performed without error. All patients performed well below ceiling but also significantly above chance (prop. correct: 1846 = 0.833; 2363 = 0.875; 2563 = 0.792; each *p* < 0.01; Figure 2B). Visual recognition performance was perfect for all participants which suggested good retention by patients but prevented formal between-group statistical tests (Figure 2C).

In the EE task, two of three patients (1846 and 2363) performed almost identically to their last-epoch CSSL performance (prop. correct: 1846, CSSL = 0.77 vs. EE = 0.75; 2363, CSSL = 0.75 vs. EE = 0.79) while patient 2563 showed a marked improvement (CSSL = 0.57 vs. EE = 0.98; Figure 2A, rightmost points). All patients performed significantly above chance (each *p* < 0.001). The secondary NC group (*N* = 4) performed without error. Thus, after EE exposure all patients had above-chance learning which was less than NC performance (albeit only slightly for 2563).

## Discussion

We found that two of three patients with bilateral hippocampal pathology were able to learn new word-object associations in a CSSL paradigm. This is consistent with the suggestion that the hippocampus is not strictly necessary for statistical learning (Covington et al., 2018)—and conversely, that non-hippocampal brain regions can support statistical learning. Our findings are also consistent with neuropsychological findings indicating that patients with amnesia due to MTL or focal hippocampal pathology can sometimes learn new word-object associations (Duff et al., 2006). Critically, our work extends those earlier findings by demonstrating that patients with hippocampal pathology can simultaneously learn multiple arbitrary, multimodal word-object associations even when potentially interfering information is presented (Roembke and McMurray, 2016). This novel finding addresses a key question regarding the necessity of the hippocampus for CSSL, and it makes contact with several current theories of hippocampal contributions to learning and memory as well as theories of statistical word learning.

### Interindividual Differences in Task Performance Within the Current Patient Group

Our finding that patients with hippocampal pathology acquired new words from CSSL includes certain caveats. Of the three patients, two (1846 and 2363) showed robust learning while the third (2563) did not. This individual difference was not obviously attributable to the degree of memory impairment, etiology, or neuroanatomy. Notably, the patient who did not show evidence of learning, 2563, adopted and later informally described a tactical approach to the task (alternating left-right responses) that did not benefit his performance. Because unsupervised learning paradigms (including our CSSL task) present no feedback, the ineffectiveness of a given tactic may never become evident to the learner. Such tactics have been found to affect performance in both human and non-human animal learning (Wasserman et al., 2015; Roembke and McMurray, 2016). We speculate that 2563’s tactic during CSSL may have interfered with the residual capacity for statistical learning shown by the two remaining patients.

This account may also address 2563’s excellent performance in the EE task. In that condition, the lack of response selection during the learning phase meant that there was no opportunity to develop or apply a tactical approach. Alternatively, the substantial differences in 2563’s performance across conditions could be attributed to an unusual vulnerability to interference, but the two other patients did not exhibit a similar susceptibility. Finally, we note that 2563’s poor performance in the CSSL task reported here was qualitatively similar to his poor statistical learning performance in Covington et al. (2018) where he showed less evidence of statistical learning than 1846 and 2363.

Regarding the CSSL exhibited by patients 1846 and 2363, both showed significant evidence of acquiring word-object associations during the task. However, their learning was less rapid and less robust than the comparison group. As with a prior report (Covington et al., 2018), we interpret the learning shown by 1846 and 2363 as a reflection of contributions from a broad network of (non-hippocampal) brain regions to statistical learning performance that has been implied by prior neuroimaging studies (Bischoff-Grethe et al., 2000; McNealy et al., 2006; Turk-Browne et al., 2009; Karuza et al., 2013).

### Word Learning in Patients With Hippocampal Pathology

Patients in our study showed evidence of learning multimodal, auditory-visual word-referent representations. While near-normal recognition performance for single items has been reported for patients with hippocampal amnesia (Ryan et al., 2000; Konkel et al., 2008), residual learning of inter-item relational information is unusual (Giovanello et al., 2003; Mayes et al., 2004; Turriziani et al., 2004; Hannula et al., 2007; Konkel et al., 2008) but not unprecedented, even in the context of word-referent representations. For example, Duff et al. (2006) tested EE of picture-word pairs in a control condition. After 24 exposures, patients showed some learning of picture-word pairs in a cued recall test (mean = 35% correct) although it was much less than that of comparison participants (who were 100% correct after only four exposures). In contrast, Warren and Duff (2014) tested word-referent learning in two conditions (EE and fast mapping (FM)] and observed no evidence of above-chance learning after two exposures. Other studies contrasting EE and FM word learning have also reported little evidence of learning multimodal relational from small numbers of exposures (for review see Cooper et al., 2019).

A key difference between studies of word-referent pairs that observed no learning and those that observed some learning may lie in the number of stimulus presentations. Duff et al. (2006) found evidence of limited but measurable multimodal relational learning after 24 presentations; here, we observed impaired but measurable learning across 42 CSSL presentations per word-object pair; and studies that reported little or no learning typically provided many fewer presentations (e.g., Warren and Duff, 2014). This suggests that the massed practice which characterizes CSSL paradigms may allow slower, non-hippocampal brain systems to learn multimodal, relational information. As with prior studies that demonstrated evidence of inefficient but measurable learning by patients with amnesia (Scoville and Milner, 1957; Cohen and Squire, 1980; Glisky et al., 1986; Glisky, 1992), our finding that multimodal word-referent representations can be learned despite hippocampal pathology suggests an intriguing translational potential for CSSL methods. However, subsequent investigations should also address why laboratory evidence for CSSL does not necessarily generalize to the ecological learning of word-referent pairs by patients with hippocampal pathology.

### Statistical Learning and the Hippocampus

Our findings contribute to a growing literature describing hippocampal contributions to statistical learning. Prior work first suggested that medial temporal lobe and/or hippocampus might make necessary contributions to statistical learning: functional neuroimaging indicated that hippocampal activation can be related to statistical learning (Turk-Browne et al., 2009; Schapiro et al., 2016); and a neuropsychological study indicated that the medial temporal lobe might be necessary for statistical learning (Schapiro et al., 2014). However, more recent work indicates that while the hippocampus may contribute to statistical learning, learning through statistical exposure is still possible despite hippocampal pathology (Covington et al., 2018) albeit reduced relative to normal performance. Our observations are consistent with the latter account, that is, the hippocampus is not strictly necessary for statistical learning—even when the statistics describe arbitrary relations between elements. Our findings also converge with neuroimaging results from Berens et al. (2018) which indicated that rapid binding of representations in the hippocampus may enhance CSSL in healthy adults.

We suggest that the nature of hippocampal contributions to statistical learning is informed by our finding that patients with hippocampal damage learned less efficiently than healthy comparison participants (see also Covington et al., 2018). Our observations are consistent with a role for the hippocampus in which it can contribute to statistical learning indirectly by supporting the rapid binding of independent and arbitrarily associated pieces of information. This familiar contribution is predicted by relational memory theory (Eichenbaum et al., 1994; Eichenbaum and Cohen, 2001), and it is consistent with our finding that healthy participants showed rapid learning of the arbitrary relations during the task. Absent contributions of the hippocampus, two patients learned some information, but that learning was slower (i.e., less learning from identical exposure; patient 1846) and/or potentially less stable (patient 2363) than healthy comparisons.

To the extent that deficits in relational memory limited performance of patients in the current word-referent learning task, we would predict that tasks that required memory for additional relations (e.g., between more items) would show similar or greater deficits in performance. A reasonable question might be, is it possible that this outcome could also obtain if the nature of the deficit was qualitatively different? If the deficits in CSSL that we observed were (for example) exclusively attributable to a hippocampus-dependent impairment in incremental learning of associations from statistical exposure, would the same outcome be observed? While not impossible, this explanation would not be consistent with substantial prior evidence that patients with hippocampal pathology can often show incremental learning as efficient as that of healthy comparison participants in a variety of laboratory tasks (Milner, 1968; Cohen and Squire, 1980; Duff et al., 2006). An important caveat is that such tasks have typically used explicit, deterministic exposure rather than incidental, statistical exposure. Our approach intentionally replicated typical CSSL methods to align with the existing literature, but future studies might be expressly designed to probe this issue. Testing the nature of the CSSL representations for hallmark features of relational representation (part-cued retrieval, flexibility, etc.) in patients and healthy comparisons would be especially informative.

### Statistical Learning and Non-hippocampal Brain Regions

Although our design was not intended to exhaustively probe patient memory representations, we speculate that patient memory representations would have hallmark features of non-hippocampal memory including contextual dependence, lack of generalizability, and inflexibility (Cohen and Squire, 1980; Glisky et al., 1986; Duff et al., 2006; Warren et al., 2012). Alternatively, our findings could also be interpreted through the lens of complementary learning systems models (McClelland et al., 1995; Norman and O’Reilly, 2003). Under this interpretation, the availability of enhanced pattern separation and completion supported by the hippocampus may have enhanced the speed of statistical learning for healthy participants by sharpening representations of studied associations (Schapiro et al., 2016). Meanwhile, non-hippocampal MTL (and other brain regions) would support slower learning that is more prone to interference because of relatively poor pattern separation (McCloskey and Cohen, 1989; Norman and O’Reilly, 2003). While we believe that the relational memory account is especially informative, our observations of less efficient learning by patients with hippocampal pathology are consistent with either perspective.

### CSSL and Hippocampus: On-Line Processing of Representations

Encoding durable memory representations is a hallmark of hippocampal function, but the hippocampus is also increasingly understood to contribute to ongoing cognitive processes (“memory at the moment”) in ways that may influence CSSL performance. Patients with hippocampal pathology have been shown to perform more poorly than healthy comparisons in a variety of tasks which do not put obvious demands on long-term memory representations such as visual search tasks (Barense et al., 2007; Voss et al., 2011; Warren et al., 2011, 2012).

This is highly relevant to CSSL because it has been hypothesized that two distinct processes comprise CSSL (Roembke and McMurray, 2016; Roembke et al., 2018): (1) a gradual associative process which incrementally updates word-referent weights; and (2) a rapid, real-time inference process employed during referent selection based on the current weightings (McMurray et al., 2012). In this framework, the second process does not reflect learning. Instead, it describes real-time processing which allows participants to combine any available evidence of (statistical) associations with the current context to make a more accurate decision. One effect of this processing may be the temporary amplification of relatively weak mappings to achieve better accuracy in the moment (Yurovsky et al., 2014).

This latter process may benefit from hippocampal-dependent processing of information in the moment. Conversely, degraded hippocampal function could contribute to impairments in the inferential process and impair CSSL performance. Our findings would be consistent with this account. Further still, statistical learning may not be simply based on the observed statistics of the input. Rather, elements of the input that receive more attention may become more strongly associated (McMurray et al., 2012; Yurovsky and Frank, 2015). From this perspective, a contribution of the hippocampus might be to strengthen associations between input elements that were preferentially attended. This would be consistent with the well-characterized roles of the hippocampus in encoding new relational representations (Eichenbaum and Cohen, 2001) and/or pattern separation (Norman and O’Reilly, 2003).

Targeted experimental designs should assess whether failures of real-time inferential processing uniquely contribute to impaired performance in patients with hippocampal pathology.

### CSSL, Hippocampus, and Language Development

Our findings are also relevant to understanding word learning during language development. We observed that the hippocampus is not necessary for learning of word-referent pairs in a CSSL paradigm. This suggests that an extended network of (non-hippocampal) language-related brain regions could support CSSL in infants and young children (Smith and Yu, 2008; Suanda et al., 2014; Fitneva and Christiansen, 2017; Vlach and DeBrock, 2017; Roembke et al., 2018). Significant word learning occurs before 36 months, a time when the hippocampus and MTL are still developing (Gogtay et al., 2006; Ghetti and Bunge, 2012; Ofen, 2012). Thus, our evidence for non-hippocampal learning suggests that other brain regions may support early developmental language milestones. This is consistent with findings from developmentally amnesic individuals with perinatal hippocampal pathology (Vargha-Khadem et al., 1997; Elward and Vargha-Khadem, 2018), as those individuals showed relatively preserved vocabulary acquisition (low-normal) despite severe deficits in declarative memory. New studies in developmental populations could test the implications of a greater childhood reliance on non-hippocampal learning by comparing the efficiency and quality of CSSL in children and adults.

## Limitations

Our study had some limitations. First, as with many neuropsychological studies, our sample size of three patients was small. This limitation did not prevent our design from capturing important information from the behavior of our sample. However, it did limit our ability to address certain questions such as a putative relationship between volume of preserved hippocampal tissue and CSSL performance. Second, the MRI exam of the hippocampus for volumetric assessment was not possible for one of the patients, but CT evaluation suggested bilateral hippocampal atrophy. While there was no evidence of atrophy of other brain regions in the patient’s CT imaging data, it is possible that his unusual pattern of chance performance on the CSSL task could be attributed to subtle neuroanatomical changes. However, this would not be consistent with his performance on standard neuropsychological tests. Third, our design could not assess the resilience or persistence of new word knowledge, although we speculate that patients would have impaired retention of new word learning over time. Retention (and consolidation) could be addressed in future research by testing learned information again after a delay. Fourth, although the CSSL and EE tasks used unique stimuli and were administered in separate sessions, the order of administration to the patient group was fixed (CSSL then EE). Meanwhile, the healthy comparison groups completed either the CSSL or EE tasks, but not both. Because healthy comparison performance was perfect in the EE task, we do not believe that they were selectively disadvantaged by the relative novelty of the task. Similarly, it is not clear how prior exposure to a different (CSSL) task would influence the EE task performance of the patient group. However, counterbalancing of task order could be used in future studies to address concerns regarding any potential confound of this nature. Finally, the CSSL task used here was subject to certain design constraints. The number of studied items was limited, the number of competitor items was fixed and small, and the word-referent pairings were deterministic (vs. stochastic). These elements of our design were deliberate and intended to provide sufficient power for our novel investigation of CSSL in patients with hippocampal pathology. Future investigations seeking to extend our findings should parametrically vary design parameters with the goal of refining the field’s understanding of hippocampal contributions to CSSL.

## Conclusions

In conclusion, our findings are consistent with the suggestion that the hippocampus is not strictly necessary for statistical learning (Covington et al., 2018). Rather, the hippocampus may contribute to CSSL by: (1) providing an additional route for faster learning; and/or (2) supporting real-time processing to improve performance at the moment. Critically, this supports accounts of CSSL that include the incremental accumulation of statistics or the gradual building of associations (in addition to more rapid forms of learning or inference; Frank et al., 2009; McMurray et al., 2012).

We speculate that non-hippocampal brain regions or structures that contribute to statistical learning may include medial temporal lobe neocortex (McClelland et al., 1995; Norman and O’Reilly, 2003) and basal ganglia (Poldrack et al., 2001; Poldrack and Packard, 2003; Poldrack and Rodriguez, 2004) among others (Bischoff-Grethe et al., 2000; McNealy et al., 2006; Turk-Browne et al., 2009; Karuza et al., 2013). CSSL may also benefit more specifically from contributions by a network of language-related brain regions including anterior and lateral temporal lobes (McNealy et al., 2006; Davis et al., 2009; Karuza et al., 2013; Warren et al., 2016). Additional functional neuroimaging and neuropsychological investigations might address this hypothesis.

Importantly, if our findings generalize to other populations with memory deficits due to hippocampal damage or dysfunction (e.g., Alzheimer’s disease, medial temporal lobe epilepsy, anti-NMDA receptor encephalitis), then those individuals should be able to learn new word-referent mappings under conditions promoting statistical learning. It remains to be determined whether the durability of information learned in this manner is different from more traditional explicit learning formats, but the translational potential of learning in a simple cross-situational statistical format is exciting. Finally, our work highlights the utility of multidisciplinary studies which combine methods and theoretical perspectives from the literature of language and memory (Duff and Brown-Schmidt, 2012) and the unique capacity of neuropsychological methods to inform the necessity of key brain regions for processes supporting memory, language, or both.

## Data Availability Statement

The datasets generated for this study are available on request to the corresponding author.

## Ethics Statement

The studies involving human participants were reviewed and approved by University of Iowa Human Subjects Office and by the Biomedical Institutional Review Board. The patients/participants provided their written informed consent to participate in this study.

## Author Contributions

All authors designed the research. DW, TR, and NC conducted the research. DW and TR analyzed the data. All authors prepared and approved the manuscript.

## Conflict of Interest

The authors declare that the research was conducted in the absence of any commercial or financial relationships that could be construed as a potential conflict of interest.
